# Assessment of the accuracy of 11 different diagnostic tests for the detection of *Schistosomiasis mansoni* in individuals from a Brazilian area of low endemicity using latent class analysis

**DOI:** 10.3389/fmicb.2022.1048457

**Published:** 2022-12-15

**Authors:** Silvia Gonçalves Mesquita, Roberta Lima Caldeira, Tereza Cristina Favre, Cristiano Lara Massara, Lílian Christina Nóbrega Holsbach Beck, Taynãna César Simões, Gardênia Braz Figueiredo de Carvalho, Flória Gabriela dos Santos Neves, Gabriela de Oliveira, Larisse de Souza Barbosa Lacerda, Matheus Alves de Almeida, Omar dos Santos Carvalho, Marina Moraes Mourão, Edward Oliveira, Rosiane A. Silva-Pereira, Cristina Toscano Fonseca

**Affiliations:** ^1^Grupo de Pesquisa em Helmintologia e Malacologia Médica, Instituto René Rachou, Fundação Oswaldo Cruz, Belo Horizonte, Minas Gerais, Brazil; ^2^Laboratório de Educação em Ambiente e Saúde, Instituto Oswaldo Cruz, Fundação Oswaldo Cruz, Rio de Janeiro, Brazil; ^3^Núcleo de Estudos em Saúde Pública e Envelhecimento, Instituto René Rachou, Fundação Oswaldo Cruz, Belo Horizonte, Minas Gerais, Brazil; ^4^Grupo de Pesquisa em Biologia e Imunologia de Doenças Infecciosas e Parasitárias, Instituto René Rachou, Fundação Oswaldo Cruz, Belo Horizonte, Minas Gerais, Brazil; ^5^Grupo de Pesquisa em Genômica Funcional de Parasitos, Instituto René Rachou, Fundação Oswaldo Cruz, Belo Horizonte, Minas Gerais, Brazil

**Keywords:** schistosomiasis, *Schistosoma mansoni*, diagnostic tests, diagnosis, latent class analysis, clinical research, sensitivity, specificity

## Abstract

**Background:**

Schistosomiasis is a parasitic disease associated with poverty. It is estimated that 7.1 million people are infected with *Schistosoma mansoni* in Latin America, with 95% of them living in Brazil. Accurate diagnosis and timely treatment are important measures to control and eliminate schistosomiasis, but diagnostic improvements are needed to detect infections, especially in areas of low endemicity.

**Methodology:**

This research aimed to evaluate the performance of 11 diagnostic tests using latent class analysis (LCA). A cross-sectional survey was undertaken in a low endemicity area of the municipality of Malacacheta, Minas Gerais, Brazil. Feces, urine, and blood samples were collected from 400 residents older than 6 years of age, who had not been treated with praziquantel in the 12 months previous to the collection of their samples. The collected samples were examined using parasitological (Helm Test^®^ kit Kato-Katz), nucleic acid amplification tests -NAATs (PCR, qPCR and LAMP on urine; PCR-ELISA, qPCR and LAMP on stool), and immunological (POC-CCA, the commercial anti-*Schistosoma mansoni* IgG ELISA kit from Euroimmun, and two in-house ELISA assays using either the recombinant antigen PPE or the synthetic peptide Smp150390.1) tests.

**Results:**

The positivity rate of the 11 tests evaluated ranged from 5% (qPCR on urine) to 40.8% (commercial ELISA kit). The estimated prevalence of schistosomiasis was 12% (95% CI: 9–15%) according to the LCA. Among all tests assessed, the commercial ELISA kit had the highest estimated sensitivity (100%), while the Kato-Katz had the highest estimated specificity (99%). Based on the accuracy measures observed, we proposed three 2-step diagnostic approaches for the active search of infected people in endemic settings. The approaches proposed consist of combinations of commercial ELISA kit and NAATs tests performed on stool. All the approaches had higher sensitivity and specificity than the mean values observed for the 11 tests (70.4 and 89.5%, respectively).

**Conclusion:**

We showed that it is possible to achieve high specificity and sensitivity rates with lower costs by combining serological and NAATs tests, which would assist in the decision-making process for appropriate allocation of public funding aiming to achieve the WHO target of eliminating schistosomiasis as a public health problem by 2030.

## Introduction

Schistosomiasis is a debilitating neglected tropical disease (NTD) that currently affects about 240 million people in the tropical and subtropical regions. *Schistosoma mansoni* is the main species causing intestinal schistosomiasis in Africa and the Americas, where it is estimated that 25 million people are at risk of infection (PAHO, 2022). In Brazil, there are more than 1.5 million people infected (Ministério da Saúde, 2019), especially in the Northeast and Southeast regions (Carvalho et al., 2018; Katz, 2018). Data from the records of the Brazilian national schistosomiasis control programme-the Sistema de Informação do Programa de Controle da Esquistossomose (SISPCE), indicate that the prevalence of schistosomiasis in Brazil between the years 2009 and 2019 ranged from 3.22 to 5.20%, with an average of 4.29% (BRASIL M da S, 2021). Although these percentages may suggest that Brazil is due to meet the World Health Organization (WHO) target of eliminating schistosomiasis as a public health problem (defined as a prevalence of heavy infections lower than 1%) by 2030 (WHO, 2021), it is likely that these data are underestimated because of the microscopy-based diagnostic method used, namely the Kato-Katz (KK) test (Siqueira et al., 2011; Weerakoon et al., 2018; Ogongo et al., 2022). The KK test consists of the microscopic observation of eggs in fecal samples (Katz et al., 1972) and has a high analytical specificity, low cost, and relatively simple execution. For these reasons, this is the method recommended by both the WHO and the Brazilian Ministry of Health for the diagnosis of intestinal schistosomiasis and use in epidemiological surveys. However, the performance of the KK test is limited by the intensity of the host infection, the daily variation of parasite egg excretion and the uneven distribution of eggs within fecal samples (Ogongo et al., 2022). This is especially critical in low endemicity settings, where the KK test may overlook 25–30% of the infected people, underestimating the true prevalence of the disease (Berhe et al., 2004; Enk et al., 2008; McManus et al., 2018).

The development of new diagnostic tests and tools has been extensively explored in the past years, and it is listed in the new WHO 2021–2030 road map for neglected tropical diseases (WHO, 2021) as one of the actions required to control and eliminate NTDs, including schistosomiasis. Ideally, in order to properly evaluate the performance of a new diagnostic test, its sensitivity, specificity, and accuracy need to be compared to a reference standard test (RST), where the latter is able to indicate the true-positive and true-negative individuals (Alonzo and Pepe, 1999). Although recommended for use by health authorities in clinical settings, the KK test is not appropriate for use as a RST, because its lack of sensitivity would influence (i.e., bias) the assessment of the accuracy of any new test(s) under evaluation.

In this context, latent class analysis (LCA) is a useful analytical technique that can circumvent the problem of an absence of an appropriate RST. LCA is a statistical method described in Walter and Irwig (1988) that enables the evaluation of new diagnostic methods when an RST is not available by estimating the infection status of an individual based on the combined results of the tests conducted in a particular group. It is a promising tool for analyzing new schistosomiasis diagnostic tests and many recent studies have relied on using this approach to assess the accuracy of new tests (Colley et al., 2013; Beltrame et al., 2017; Clements et al., 2017; Fuss et al., 2018; Koukounari et al., 2021). In order to address the need to improve schistosomiasis diagnosis, our study evaluated the sensitivity, specificity, and accuracy of 11 diagnostic tests using different samples (feces, urine, and blood) from 400 residents of a low endemicity area in Brazil (the municipality of Malacacheta) using the occurrence of infection estimated by LCA as a reference standard. Our findings allowed us to propose a 2-step diagnostic process, with an overall accuracy of 94%, involving the combination of serological and nucleic acid amplification tests (NAATs) for the active search for infected people in moderate and low prevalence settings.

## Materials and methods

### Ethics statement

This study followed the guidelines and regulations for clinical research—Resolution 196/1996 of the Brazilian National Health Council (CNS)—and is in line with the principles of the Declaration of Helsinki. The study was approved by both: (i) the Ethical Committee of the René Rachou Institute (IRR, Fiocruz Minas), CAAE 76273317.3.0000.5091, and approval numbers 2.400.880, 2.803.752, 3.802.104 and 3.918.849; and (ii) the Ethical Committee of the Oswaldo Cruz Institute (IOC, Fiocruz), CAAE 76273317.3.3001.5248, and approval number 2.426.395. For Ethical reasons, all participants of this study received the results of the parasitological diagnosis, and those with a positive KK result were treated using praziquantel tablets (Farmanguinhos/Fiocruz, Rio de Janeiro, RJ, Brazil), with a 60 mg/kg single dose, as recommended by the Brazilian Health Ministry (Brasil, 2014). As the KK test also allows the detection of soil-transmitted helminths (STH), the participants who had STH eggs in their feces received two albendazole tablets (400 mg) to be taken with a 15-day interval.

### Study area

The study was conducted in the borough of Santa Rita (17°50′33”S; 42°4′22″W), in the city of Malacacheta, in the state of Minas Gerais, Brazil, between the 16th of October 2018 to the 16th of November 2018. The city of Malacacheta has a high percentage (17.3%) of extreme poverty and was considered by the Brazilian Ministry of Health as one of the 222 municipalities requiring priority action for schistosomiasis control (Portaria Ministerial no 1.556 de 28 de outubro de 2011). According to the data collected by the Brazilian national schistosomiasis control programme, the Programa de Controle da Esquistossomose (PCE), coordinated by the Brazilian Ministry of Health, 7,114 diagnostic tests were carried out in Malacacheta between 2010 and 2012, of which 1,646 (23.1%) were positive, among them 105 (6,4%) having high-intensity infection (http://tabnet.datasus.gov.br/cgi/tabcgi.exe?sinan/pce/cnv/pcebr.def, accessed on 04/25/2022). A school survey performed in 2013, based on two KK slides per stool sample, indicated an estimated prevalence of infection of 24.3% among schoolchildren (6–15 years; Favre et al., 2021). Data provided by a Basic Health Unit (BHU) in Malacacheta (personal communication) indicated a rise in the number of schistosomiasis cases among the inhabitants of the borough of Santa Rita during 2017. This, together with the socio-economic and environmental vulnerability of the residents, determined the choice of Santa Rita as the study area.

### Study design

The current cross-sectional study was designed to assess by LCA the accuracy of parasitological, NAATs, and immunological methods for schistosomiasis diagnosis employing different biological samples (feces, blood, and urine). The study followed the recommendations of STARD 2015 (Bossuyt et al., 2015), and all the procedures were blindly performed by experienced members of the team supervised by a senior researcher according to Good Clinical Practices. The STARD 2015 checklist is available on Supplementary material 1.

#### Inclusion criteria

The target population was the residents of the borough of Santa Rita who both expressed an interest in joining the study and met the following eligibility criteria: (i) being resident in Santa Rita, (ii) being older than 6 years of age, (iii) willing to voluntarily provide blood, urine, and fecal samples, (iv) to have not received treatment for schistosomiasis in the previous 12 months prior to sample collection, and (v) signing the Informed Consent Form (ICF) and/or the Informed Term of Consent (ITC).

A total of 410 residents of the borough of Santa Rita were included in the study after the recruitment step, which was conducted at the local BHU Waldemar José Pereira. All the participants received unique ID numbers (from 001 to 410) to preserve their confidentiality. Ten individuals were excluded from the study either due to: one person had a previous treatment with praziquantel, one participant did not agree to provide one of the biological samples during the recruitment process, eight individuals did not deliver one of the three types of biological sample during the collection or delivery process.

#### Sample size calculation

The sample size used was calculated using epiDisplay package in the R software considering the sensitivity of the test with the lowest sensitivity (i.e., the KK test) among all the tests used in the current study the script below:

a < −n.for.survey(p = sens,delta = 0.10).

a

n < −ceiling(ceiling(a$n1)/p).

n

where, sens (sensitivity for the KK test) was 0.57; n.for survey (number of inhabitants of Santa Rita borough) was 1421; p (estimated prevalence of disease) was 0.281.

Thus, a sample size of 410 individuals was inferred as being required from a total population size of 1,421 residents, calculated assuming a 57% test sensitivity for Kato-Katz, a prevalence of infection of 28.1%, a 95% confidence level, a maximum marginal error of 10%, plus a 20% loss of enrolled participants during follow-up (Eldridge et al., 2006).

**Figure 1:**
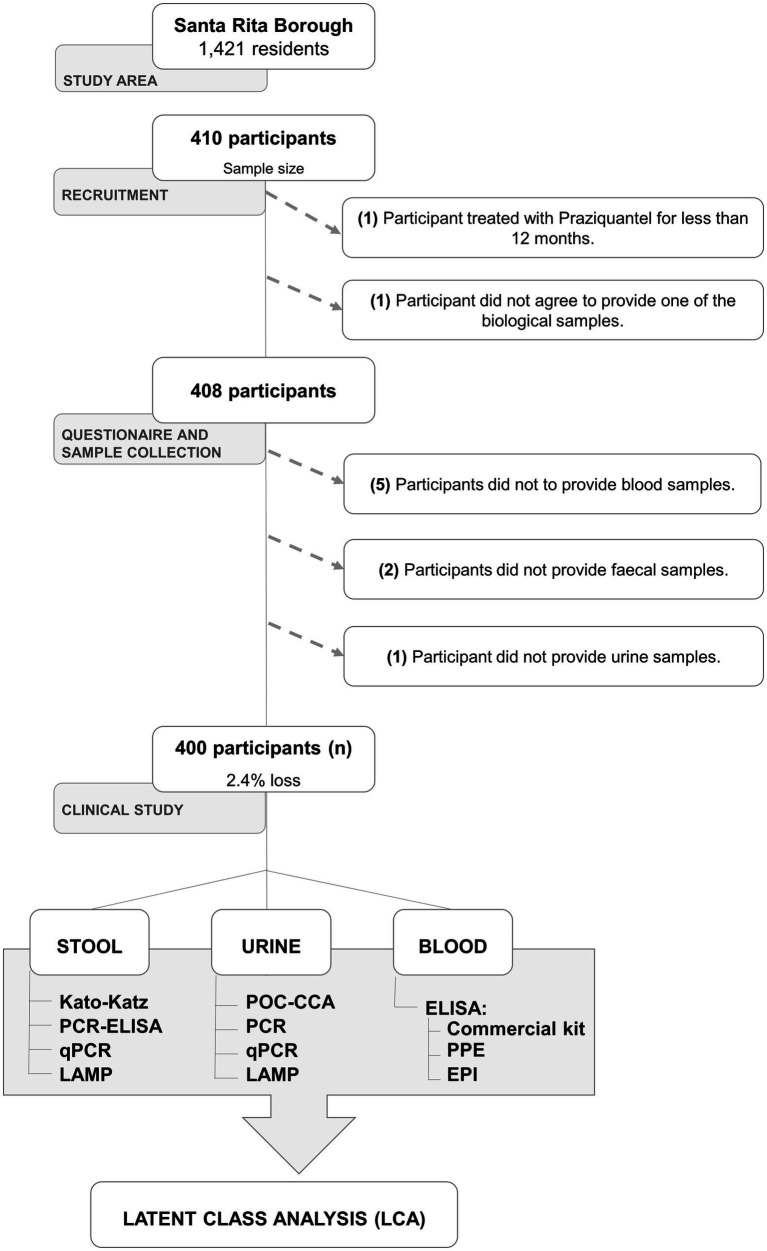
Flowchart detailing the study design. Legend: PCR-ELISA = polymerase chain reaction coupled with the enzyme-linked immunosorbent assay; qPCR = quantitative real-time PCR; LAMP = loop-mediated isothermal amplification; POC-CCA = point-of-care circulating cathodic antigens; Commercial kit = anti-*Schistosoma-mansoni* IgG commercial ELISA kit from Euroimmun; PPE = in-house anti-IgG ELISA test using the recombinant antigen PPE; EPI = in-house anti-IgG ELISA test using the Smp150390.1 peptide.

The sample size calculation described above, which was performed before the study was started, assumed a loss of 20% of the enrolled participants. However, the actual loss of enrolled participants was less than 2.4%. Therefore, our study was conducted with 400 participants, and its statistical power was not affected by loss of enrolled participants (Figure 1).

#### Sociodemographic questionnaire and history of schistosomiasis

At the BHU Waldemar José Pereira, after having a 10 ml sample of blood collected, the participants answered a questionnaire. The following information was obtained: age, gender and history of previous infection. The history of the schistosomiasis was assessed through questions concerning how many diagnostic tests had been previously performed prior to the current study (and, if any, what kind of biological material was used and what was the result of the test), and whether praziquantel was ever taken. The questionnaire was filled out by qualified research staff, based on the information provided by the participants. At the end of this phase of the study, the participants received containers for the collection of urine and feces together with the recommendations for the collection of the samples. Those samples were later handed to the Community Health Agents (CHAs) during home visits.

### Sample collection

A temporary laboratory was set up at the Secretariat for Education building, which had four workstations, each exclusively used for one of the following: (i) stool sample preparation and slide reading for the KK test; (ii) blood sample preparation; (iii) urine sample preparation; (iv) POC-CCA execution. The blood samples were collected at the Basic Health Unit on the same day the questionnaire was conducted. Urine and stool samples were collected by the participant, preferably on the same day although a 7-day interval was allowed between the collection of each sample. Stool and urine samples were delivered by the participant to a team member that went house-to-house to get those samples. If the urine and stool samples were not provided within this 7-day period, the participant was subsequently removed from the study population.

#### Blood samples

Blood samples (10 ml) were collected from the participants using appropriately labelled BD vacutainer tubes. The tubes were transported to the temporary laboratory, where they were kept at room temperature for at least 1 h after obtaining the blood samples to allow complete clot formation and then centrifuged at 1000–1300×*g* at room temperature for 10 min to obtain the serum, which was aliquoted into cryogenic tubes and stored in the freezer at-70°C until use.

#### Fecal samples

Fecal samples were collected in appropriately pre-labelled containers and were processed on the same day of collection for parasitological and NAATs. The samples were separated into two portions: (i) ~84 mg for the preparation of two KK slides (~42 mg each), and (ii) ~500 mg cryopreserved to be used later for DNA extraction and molecular tests. The 500 mg portion was separated using 3D-printed models provided by the 3D Print Facility of the IRR.

#### Urine samples

The participants collected at least 35 ml of their first-morning urine. At the workstation, the samples underwent a chemical analysis using Combur10 Test^®^ M test strips (Roche, Basel, Switzerland) to check and record any alterations in pH value, and detect the presence of leucocytes, protein, hemoglobin, and/or nitrite. This step was conducted by two experienced researchers simultaneously. For that, a test strip was immersed into every urine sample for about 1 s and then, after 1 min, the color presented on each pad of the strip was compared to the reference scale. The interpretation was conducted according to the manufacturer’s instructions, and the Guidelines from the Brazilian Society for Clinical Pathology and Laboratory Medicine (SBPC/ML; Andriolo et al., 2017). After that, two drops of each urine sample were used for the POC-CCA test. The remaining volume was filtered in a cone folded Whatman^®^ qualitative filter paper, Grade 3:6 μm (diameter 185 mm), previously labelled with the participant ID. After the filtration, the paper filters were left exposed on a sterilized bench to completely dry. They were then individually stored in hermetically sealed plastic bags, together with desiccant silica gel, and then transported at room temperature from the field to the laboratory, where the DNA extraction and NAATs tests were conducted.

### Kato–Katz test

Two slides were prepared, each with ~42 mg of feces, as recommended by the Brazilian Ministry of Health. In order to estimate the parasite load of each participant, the instructions of the Helm Test^®^ Kit (Bio-Manguinhos/Fiocruz, Rio de Janeiro, RJ, Brazil) were followed, such that the total number of eggs found on each of the two slides were added together and divided by 12 to calculate the number of eggs per gram of stool (eggs/g). The slides were read 3 h after preparation. For quality control, 10% of the slides were read by two technicians, and a third technician read 10% of the total slides of this study. In all instances, the technicians examined the slides “blind” and did not know the results when others had already read the same slides.

### POC-CCA test

The POC-CCA (Urine CCA (Schisto) ECO test) was performed to detect the presence of the *S. mansoni* circulating cathodic antigen in fresh urine. When this was not possible, the samples were refrigerated until the next day. In this case, prior to testing, the urine was removed from the refrigerator and held at room temperature for up to 2 h before testing. The urine samples were tested by Urine CCA (Schisto) ECO test (Eco Diagnóstica, Nova Lima, MG, Brazil-product reference: TR.0301CA020, batch number: 201806011, expiration date: 01/31/2021), following Eco Diagnóstica’s instructions for transport, storage, and use. After preparing a panel of properly labelled urine samples, with respective cassettes labelled with each participant’s ID. Each sample was homogenized, and two drops of urine were transferred to the cassette. After the 21^st^ minute, the result was interpreted by technicians who were unaware of the stool test results. At the time of reading, all tests were photographed, and these records were kept in the study files. According to the manufacturer, valid tests were categorized as negative, trace, or positive, with trace being considered as a positive result. Non-valid tests were repeated until a valid result was obtained.

### Nucleic Acid Amplification tests

#### Source of samples used as positive controls for NAATs

Genomic DNA (gDNA) extracted from *S. mansoni* adult worms (LE strain) using the Wizard^®^ Genomic DNA Purification Kit (Promega, Madison, WI, United States), following the manufacturer’s protocol, was used as positive controls in all NAATs performed. The gDNA concentration was determined using Nanodrop Spectrophotometer (Thermo Fisher Scientific, Waltham, MA, USA) and diluted to working concentrations of either 1 ng/μl (for LAMP) or 5 ng/μl (for qPCR, PCR and PCR–ELISA). A schematic demonstration of molecular tests is illustrated in Supplementary material 2.

#### DNA extraction

##### DNA extraction from fecal samples

The gDNA from 400 fecal samples was extracted using three different commercial nucleic acid extraction kits: (i) QIAamp DNA Stool Mini Kit (Qiagen GmbH, Hilden, Germany), according to the manufacturer’s protocol “DNA Isolation from Stool for Pathogen Detection and DNA Isolation from Large Amounts of Stool”; (ii) QIAamp Fast DNA Stool Mini Kit (Qiagen GmbH); and (iii) QIAamp PowerFecal Pro DNA Kit (Qiagen GmbH). Although the latter kit is designed to extract the DNA from 250 mg of stool, we used samples consisting of 500 mg of stool. However, the protocol was still effectively performed as recommended by the manufacturer by splitting each 500 mg fecal sample into two 250 mg halves and performing separately the “Experienced User Protocol” on each “half” until step 7. The two “halves” were then added (one at a time) to the MB Spin Column, and the remaining steps of the protocol followed treating the two pooled halves as a single sample (Qiagen, personal communication).

##### DNA extraction from urine samples

The gDNA from 400 urine samples was extracted using the commercial QIAamp DNA Blood Mini Kit (Qiagen GmbH, Hilden, Germany). Twelve circular pieces of 6 mm each were punched from every paper filter using a hole puncher. In order to avoid cross-contamination, the hole puncher was sterilized with ethanol 75% after each use. For each sample, all pieces of filter paper were transferred to a single 1.5 ml tubes with 600 μl nuclease free water and heated at 95°C for 10 min. The tubes were then incubated for 16–18 h at 22–25°C (Lodh et al., 2017). Up to 400 μl were transferred to a new labelled 2 ml tube, which was used for the DNA extraction following the manufacturer’s protocol “Purification from Blood or Body Fluids (Spin Protocol).”

### PCR-ELISA assay

A highly repetitive genome sequence of parasites from the genus *Schistosoma* (Gen Bank M61098) was amplified by PCR following the protocol described by Gomes et al. (2009, 2010) with a few modifications. Briefly, PCR was performed using a final volume of 20 μl, containing 2 μl of 10X PCR buffer, 2 μg of BSA (Sigma Aldrich, Saint Louis, MO, USA), 0.5 μM of each *S. mansoni*-specific primer (Table 1), 1.5 mM MgCl_2,_ 200 μM of each dNTPs (Promega, Madison, WI, USA), 2.0 U of Platinum™ Taq DNA Polymerase (Thermo Fisher Scientific), and 2 μl of gDNA purified from stool samples diluted 1:5 in linear acrylamide solution [30 μg/ml (*w*/*v*)]. The cycling programs were preceded by 12 min at 95°C, and then:15 cycles of 95°C for 1 min, 63°C for 1 min and 72°C for 30 s; 12 cycles of 80°C for 1 min, 63°C for 1 min and 72°C for 30 s; and 7 cycles of 80°C for 1 min, 65°C for 1 min and 72°C for 30 s; followed by a final elongation step at 72°C for 7 min. In each PCR assay, a negative control (PCR mix without DNA) and a positive control (gDNA from *S. mansoni* adult worms) were included. The presence of amplicons was detected in MaxiSorp^®^ polystyrene microplates (Nunc^™^, Thermo Fisher Scientific) following the protocol described by Senra et al. (2018). The primers used are detailed in Table 1. The human *β-actin* (*ACTB*) gene was used as an internal control, and negative (PCR mix without DNA) and positive (using DNA extracted from adult *S. mansoni* worms as template) controls were both included. The cut-off of the PCR-ELISA used in this study was 0.136, as previously defined using a receiver operating characteristic curve (ROC Curve) analysis (Senra et al., 2018). This test was performed only on stool samples.

**Table 1:** Primers and probes used in the molecular tests.

Type	Amplicon	Target	Assay	Sequence	References
Forward Primer	121 bp	*S. mansoni* repetitive region *Sm1-7* (GenBank: M61098)	PCR and PCR-ELISA	5-Biosg/ GAT CTG AAT CCG ACC AAC CG-3′	Gomes et al. (2009)
Reverse Primer				5′- ATA TTA ACG CCC ACG CTC TC-3′	
Probe				5-6[FAM]/ TGG TTT CGG AGA TAC AAC GA-3′	
Forward Primer	120 bp	Human *β-actin* gene (GenBank: AY582799.1)	PCR-ELISA	5-Biosg/ ACC TCA TGA AGA TCC TCA CC-3’	Musso et al. (1996)
Reverse Primer				5′- CCA TCT CTT GCT CGA AGT CC-3′	
Probe				5-6[FAM]/ TCT CCT TAA TGC ACG CAC G-3′	Gomes et al. (2010)
Forward primer	90 bp	*S. mansoni* repetitive region *Sm1-7* (GenBank: M61098)	qPCR	5′-CCG ACC AAC CGT TCT ATG A-3′	Siqueira et al. (2021)
Reverse Primer				5′-CAC GCT CTC GCA AAT AAT CTA AA-3′	
Probe				5′-6[FAM]/TCG TTG TAT CTC CGA AACCAC TGG ACG/[3BHQ1]	
Forward Primer	92 bp	Human *β-actin* gene (GenBank: AY582799.1)		5’-CCA TCT ACG AGG GGT ATG-3’	
Reverse Primer				3′-GGT GAG GAT CTT CAT GAG GTA-5’	
Probe				5′- 6[JOE]/CCT GCG TCT GGA CCT GGC TG/[3BHQ1]	
Internal forward Primer- FIP	NA	Mitochondrial *S. mansoni* minisatellite DNA region (GenBank: L27240)	LAMP	5′- GCC AAG TAG AGA CTA CAA ACA TCT TTG GGT AAG GTA GAA AAT GTT GT-3’	Fernández-Soto et al. (2014)
Internal Backward Primer- BIP				5′- AGA AGT GTT TAA CTT GAT GAA GGG GAA ACA AAA CCG AAA CCA CTA-3’	
External Forward Primer- F3	203 bp			5′- TTA TCG TCT ATA GTA CGG TAG G-3’	
External Backward Primer- B3				5′- ATA CTT TAA CCC CCA CCA A-3’	

bp = base pair; FAM = 6-Carboxyfluorescein; BHQ1 = Black Hole Quencher 1; JOE = 6-carboxy-4′,5′-dichloro-2′,7′-dimethoxyfluorescein; NA = not applicable.

### PCR

PCR assays were conducted as described above in Section 7.3 using 2 μl of gDNA from urine samples diluted 1:2 in linear acrylamide solution [30 μg/ml (*w*/*v*)] as template for each reaction. The results were visualized by electrophoresis using 6% polyacrylamide gels and analyzed by silver staining.

### qPCR assay

The quantitative PCR (qPCR) reaction was performed according to Siqueira et al. (2021). Briefly, the reaction was done in a final volume of 20 μl containing 10 μl of TaqMan^®^ Universal PCR Master Mix (Life Technologies, Thermo Fisher Scientific), 0.1 μM of each *S. mansoni*-specific primer, 0.25 μM of the *S. mansoni*-specific probe, 0.15 μM of each *β-actin* specific primer and 0.25 μM of the *β-actin* probe (Table 1), 2 μg of BSA, 2 μM MgCl_2_, and 4 μl of gDNA extracted from stools and diluted 1:5 in linear acrylamide solution [30 μg/ml (w/v)]. Two controls were used for each reaction: a positive control (PCR mix plus gDNA from adult worms) and a negative control consisting of PCR mix only (i.e., no template control). All primers and probes used are listed in Table 1. The assays were performed in duplicate using microplates (MicroAmp^®^ Fast Optical-Applied Biosystems, Foster City, CA, United States) sealed with adhesive film (Optical Adhesive Covers-Applied Biosystems, Foster City, CA, USA) on the StepOnePlus^™^ Real-Time PCR System (Thermo Fisher Scientific) under the universal cycling program with 45 cycles and annealing temperature of 60°C. Samples with observed Ct ≤ 42 were classified as positive, according to Siqueira et al. (2021). Samples that did not have amplification of the internal control (i.e., amplification of the *β-actin* gene) were retested, and a new DNA sample was extracted when necessary. For the urine samples, the reaction was done in a final reaction volume of 20 μl following the protocol described above, but with the follow modifications: 2.0 mM MgCl_2_ and 4 μl of DNA diluted 1:3 in linear acrylamide solution [30 μg/ml (*w*/*v*)] were used. Additionally, for the urine sample, the Ct cut-off used was ≤ 44 defined through a standard curve analysis that showed amplification up to 0.38 fg of *S. mansoni* DNA. Samples that did not have amplification of the internal control gene were retested and a new DNA sample was re-extracted when necessary.

### Lamp

The primers designed by Fernández-Soto et al. (2014) targeting the mitochondrial *S. mansoni* minisatellite DNA region (GenBank: L27240) were used in this study (Table 1). The original protocol described by the authors was adapted to ensure the specificity of the reaction. The adapted protocol consisted of a final volume of 25 μl having: 1 × Isothermal Amplification Buffer (20 mM Tris–HCl, 10 mM (NH4)_2_SO_4_, 50 mM KCl, 2 mM MgSO_4_, 0.1% Tween-20, pH 8.8 @ 25°C; New England Biolabs, Ipswich, MA, United States), 6 mM MgSO_4_ (New England Biolabs), 1.4 mM of each dNTP (Invitrogen, Waltham, MA, United States), 40 pmol/μl of the internal primers FIP and BIP, 5 pmol/μl of the external primers F3 and B3, 1 M betaine (Sigma Aldrich), 8 U *Bst* 2.0 Warm-Start DNA polymerase (New England Biolabs), 2 μl of the DNA extracted from fecal samples or 5 μl of the DNA extracted from urine samples. The reaction tubes were incubated at 63°C for 50 min, followed by a 5-min incubation at 80°C to inactivate the polymerase. The result was visualized by naked eye after the addition of 2 μl of the DNA intercalating dye SYBR Green I (1000X, Life Technologies, Thermo Fisher Scientific). When positive, the reaction changed color from orange to yellow. When negative, the reaction remained orange. The reaction tubes were also exposed to UV light (320 nm) and the samples were considered positive when the reaction tube showed fluorescent signal, whilst the absence of amplification was inferred when no fluorescence was apparent. As a quality control, 3 μl of the reaction product was visualized on silver-stained 6% polyacrylamide gel. If the result observed by adding the SYBR Green I dye and by electrophoresis did not match, the reaction was repeated just once. After repeating, any discordant results were resolved by considering visual inspection of any color changes.

### Serological tests

#### *Schistosoma mansoni* antigens

A synthetic peptide (SLPSNAHNNDNNSSD-biotin) containing amino acids 216–230 from the *S. mansoni* protein Smp150390.1 (Carvalho et al., 2017) was purchased from Biomatik (Ontario, Canada) and conjugated with biotin at the C-terminal end. The synthetic peptide had a purity of 96.22% and was resuspended in ultra-pure water to a final concentration of 2.5 mg/ml and stored at-70°C until use. A *S. mansoni* recombinant protein, called by the name PPE (rPPE), and encoded by a sequence similar to the *Smp_049300.3* gene, was expressed in *E. coli* ArcticExpress (DE3) cells (Agilent Technologies, Santa Clara, CA, United States) using the pET21a plasmid and purified by Ni^2+^ affinity chromatography using the Ni-NTA Fast Start Kit (Qiagen GmbH, Hilden, Germany). The resulting purified rPPE was stored at-70°C in PBS at 1 mg/ml until use.

#### ELISA tests

Three different ELISA tests were performed using sera from the participants of the study: the anti-*Schistosoma mansoni* IgG commercial ELISA Kit from Euroimmun (São Caetano do Sul, SP, Brazil), and two in-house ELISA tests using either the biotin-labelled synthetic Smp150390.1 (216–230) peptide or the recombinant PPE protein as antigens, respectively.

The anti-*Schistosoma mansoni* IgG ELISA test from Euroimmun (from here on in termed the commercial ELISA kit) was performed following the manufacturer’s instructions using the buffers provided in the Kit. Briefly, microtiter plates adsorbed with *S. mansoni* purified soluble egg antigens were incubated with serum samples diluted 1:101 in sample buffer for 1 h at 37°C. The plates were washed four times with a wash buffer and the detection antibody (anti-human IgG-HRP) was then added and incubated for 30 min at 37°C. After an additional washing step, color reactions were developed by incubating the substrate for 30 min at room temperature. The reaction was stopped using the stop solution and the absorbance was measured at 450 nm using an ELISA microplate reader (Thermo Fisher Scientific). Sample reactivity was determined by the ratio between sample and calibrator absorbances. Values below 0.8 were taken to indicate non-reactive sera, while values higher than or equal to 1.1 indicated reactive sera. Values higher than or equal to 0.8, but lower than 1.1, were interpreted as indeterminate results.

Anti-IgG EPI (ELISA test to detect reactivity against the synthetic peptide) containing the epitope Smp150390.1 (216–230) and anti-IgG PPE ELISA tests (ELISA test to detect reactivity against the recombinant protein PPE), from here on in termed “ELISA EPI” and “ELISA PPE,” respectively, were performed as follows. MaxiSorp 96-well microtiter plates (Nunc^™^, Thermo Fisher Scientific) were coated with either 25 μg/ml (synthetic peptide) or 8 μg/ml (rPPE) diluted in carbonate–bicarbonate buffer 0.05 M, pH 9.6, for 16 h at 4°C. The plates were blocked for 2 h at 37°C with 300 μl/well of PBS-T (phosphate-buffered saline, pH 7.2 with 0.05% Tween-20) with 10% FBS (fetal bovine serum; GIBCO, United States). One hundred microliters of each serum sample diluted either 1:40 (anti-IgG EPI test) or 1:100 (anti-IgG PPE test) in PBS-T was added per well and incubated for 2 h at 37°C. Serum samples were analyzed in duplicate. Plate-bound antibody was detected using 100 μl/well of a peroxidase-conjugated anti-human IgG (Sigma Aldrich) diluted 1:40,000 (anti-IgG EPI test) or 1:60,000 (anti-IgG PPE test) in PBS-T. Plates containing the detection antibody were incubated for 2 h at 37°C. Color reactions were developed by adding 100 μl of TMB (3,3′,5,5′-Tetramethylbenzidine) substrate (Sigma Aldrich) for 30 min at room temperature. The reaction was stopped with 50 μl of 2 M sulfuric acid solution, giving 4 N (4 Normal), per well, and the absorbance was measured at 450 nm using an ELISA microplate reader (Thermo Fisher Scientific). Sample reactivity was determined by the ratio between the mean sample absorbance and the mean calibrator (pool of sera samples from non-*Schistosoma mansoni* infected individuals) absorbance. Values below 0.95 were taken to indicate non-reactive sera and values higher than 1.05 indicated reactive sera. Values higher than or equal to 0.95, but lower than or equal to 1.05, were interpreted as indeterminate results. The ranges for classification of the serum reactivity were determined based on the inter- and intra-test coefficient of variation observed during the standardization process. A schematic demonstration of ELISA tests is illustrated in Supplementary material 2.

Sera presenting indeterminate results in any of the ELISA tests were evaluated again, and the second result registered in the study database. For the construction of the latent class model, indeterminate results were considered as negative results.

### Statistical analysis

Initially, samples were characterized based on the proportional distribution of individuals in relation to the history of infection with the parasite and any previous treatment. The latent class analysis (LCA) can be used in situations where the reference standard is partially unavailable or imperfect. The LCA method combines multiple test results in order to construct a standard reference outcome. The probability of each individual being classified as ‘case’ (classified as positive for *S. mansoni* infection) based on the 11 tests described above was estimated from the fit of the latent class model (LCM), which may include a random effect (Beath, 2017). The best model in terms of the number of classes into which individuals are classified was chosen based on the lowest value of the Bayesian Information Criterion (BIC). The posterior class probabilities for each observed pattern and class were determined. These were returned as a data frame together with the patterns for each observation. Thus, the outcome of interest could be defined. Individuals who presented a posterior class probability above 80% in the best fit LCM were considered as belonging to the ‘case group’. Separate analyses of the individual accuracy of each of the 11 diagnosis tests were also performed. The 80% cut-off was defined based on the distribution of outcome probabilities (Supplementary material 3) in which there is a sharp decrease below the cut-off in the probability of being included in the ‘case group’ (from 81 to 68%).

Diagnostic test accuracy (DTA) is defined as the proportion of all the clinical diagnostic tests analyzed that give a correct result. The most common accuracy measures are: (i) sn = sensitivity, the probability of a positive test result in people with the infection; (ii) sp. = specificity, the probability of a negative test result in people without the infection; (iii) PPV = positive predictive value, the ratio of people with the infection to those who have a positive test result; (iv) NPV = negative predictive value, the ratio of people without the infection to those who have a negative test result; (v) LR+ = positive likelihood ratio, the ratio of the probability of a positive test result among those with the infection to that of a positive test result among those without the infection; (vi) LR- = negative likelihood ratio, the ratio of the probability of a negative test result among those with the infection to that of a negative test result among those without the infection; (vii) accuracy, the combined proportion of people who are true positives and true negatives among all the subjects tested. The LR is an overall measure of the discrimination of test result. The test is useless if LR = 1. The test is better the more LR differs from 1, that is, greater than 1 for LR+ and lower than 1 for LR-. The *Epi* package was used in the R software (epi.tests function) computes true and apparent prevalence, sensitivity, specificity, positive and negative predictive values, and positive and negative likelihood ratios from count data provided in a 2 by 2 table (Carstensen et al., 2022). The exact binomial confidence limits were calculated for test sensitivity, specificity, and positive and negative predictive values (Collett, 2002). Confidence intervals for positive and negative likelihood ratios were based on the formulae provided by Simel et al. (1991).

In order to calculate the summary statistics for the DTA, a pairwise meta-analysis model was selected. General pairwise meta-analysis calculates the effect size, such as relative risk and the odds ratio (OR) for binary data, and the mean difference for continuous data. DTA simultaneously combines two effect sizes, such as the sensitivity and specificity, or the positive predictive and negative predictive values. The bivariate model assumes a binomial distribution that directly models the sensitivity and specificity for within study variations, while assuming a bivariate normal distribution for between-study variation. “Different studies” were considered as different exclusive sets of tests grouped together according to the type of biological material they examined (i.e., urine, stool, or blood; Shim et al., 2019).

For calculating the confidence interval, the Clopper–Pearson method was used (Newcombe, 1998). In forest plots, the Higgins’ *I^2^* of the heterogeneity was determined by subtracting the number of degrees of freedom from the Cochrane *Q* statistics, and then again dividing the resulting value by the Cochrane *Q* statistics. Thus, it quantifies the heterogeneity in a consistent manner. Heterogeneity may be suspected if the between-study variation is greater than the within-study variation in the forest plot (sensitivity and specificity). Methods for meta-analysis of fixed effects and random effects of single proportions were used to calculate an overall proportion (Borenstein et al., 2010; Barendregt et al., 2013). The packages in R software used were *random LCA*, *Epi*, *metaprop* and *metabin* for sensitivity, specificity, and diagnostic odds ratio; and *forest* for the forest plot (Beath, 2017; Balduzzi et al., 2019; Carstensen et al., 2022).

### Rationale for calculating the cost of tests

The cost of each test assessed in this study was calculated according to the current price quoted in Brazilian reais (BRL) between June and July 2022. The exchange rate for American dollars (USD) was obtained from the Central Bank of Brazil (https://www.bcb.gov.br/) on 27/07/2022, when 1 USD = 5.3068 BRL. The costs of the 2-step protocols proposed in the present study were calculated using the following formulas:


n×cost of comercial ELISAkit=X

nPOS×cost of molecular testonstool=Y

$$\begin{array}{l} \mathrm{cost}\;\mathrm{per}\;\mathrm{individual of the proposed}\;\mathrm{two}-\mathrm{step protocol}\\ =\left(X+Y\right)÷n \end{array}$$

When *n* is the population size and *nPOS* is the number of individuals classified as positive by the commercial ELISA kit.

The cost for each sample does not include the price of equipment needed, personnel, and DNA extraction kits (for NAATs). The cost of DNA extraction kits is listed separately on Supplementary Material 4. The price of the discontinued QIAamp DNA Stool Mini Kit, the KK and POC-CCA kits were obtained previously (2017–18) and adjusted according to the General Market Price Index (IGP-M) from Getúlio Vargas Foundation (https://portal.fgv.br/). The calculation of the costs is described in detail in Supplementary Material 4.

## Results

### Demographic data

The participants answered a questionnaire enabling the determination of their demographic profile. The age of the study population ranged from 6 to 88 years, with 51.5% of participants with less than 30 years of age. The majority were female (65.5%). More than 56% of the participants had schistosomiasis at least once in their lives. All the demographic data collected by the questionnaire are detailed in Table 2. No adverse effects associated with either sample collection or treatment for worm infection were observed.

**Table 2:** Summary of the demographic data of the study population (*N* = 400).

Population characteristics	*n* (400)	%
Age		
6 - 8	35	8.8
9 - 11	40	10.0
12 - 14	38	9.5
15 – 29	93	23.2
30 – 47	95	23.8
48 - 88	99	24.8
Gender		
Female	262	65.5
Male	138	34.5
Previously tested for schistosomiasis		
Yes	258	64.5
No	130	32.5
Did not know	12	3.0
Among those previously tested	***n* (258)**	**%**
Biological material used for previous testing		
Faeces		
Blood	252	97.7
Blood and Faeces	4	1.6
Did not know/ Did not answer	2	0.01
	4	1.6
Previous test result		
Positive	145	56.2
Negative	97	37.6
Did not know	16	6.2
Previous treatment with praziquantel		
Yes	130	89.7
No	15	10.3
Time since previous treatment		
> 1 year	6	4.6
> 3 years	124	95.4

### Kato-Katz technique

**Figure 2:**
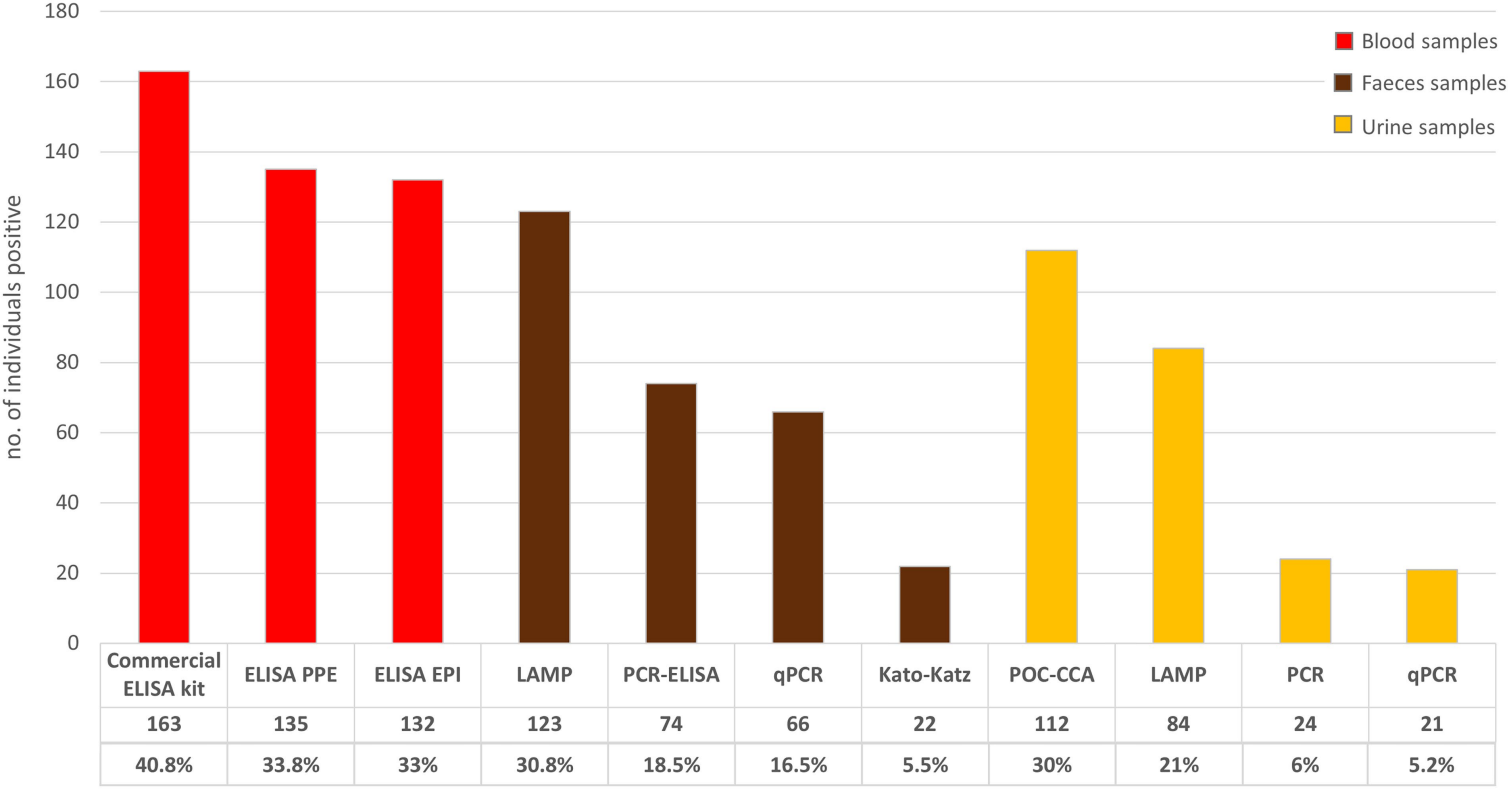
Detection of schistosomiasis infection according to different diagnostic tests and sample types. POC-CCA positive results include trace outcome and serologically negative results include indeterminate results.

The KK technique enabled the detection of 22 individuals (5.5%) having *S. mansoni* eggs in their stools (Figure 2). Based on the egg count per gram of stool, the distribution of the intensity of *S. mansoni* infection was as follows: 81.8% of *S. mansoni*-infected participants had light infections (1–99 eggs/g), 9.1% of moderate infections (100–399 eggs/g), and 9.1% heavy infections (> 400 eggs/g; Gomes et al., 2017). Besides the detection of *S. mansoni* infection, KK also enables the detection in stools of the eggs of soil-transmitted helminths (STH). In this study, the presence of the nematode *Ascaris lumbricoides* was detected in six individuals (1.5% of all participants), and unspecified hookworms in one individual (0.2%). The occurrence of multiple infections (i.e., simultaneous coinfection of the same individual with both *S. mansoni* and STH) was not detected.

### Immunological tests

POC-CCA identified 122 (30.5%) individuals as positive for the presence of circulating cathodic antigen (when also including the trace outcome; Figure 2). Serological tests were conducted to detect the presence of IgG antibodies in the sera of the study population. The commercial ELISA kit classified 163 individuals as positive (40.8% of the total study population), while 53% (212/400) had a non-reactive serum, and the remaining 6.3% (25/400) had indeterminate results, with both of the latter groups being considered negative for the purposes of the current study. A positivity rate of 33% (132/400) was observed when the ELISA EPI was performed, with 65.8% (263/400) of samples non-reactive and 1.3% (5/400) indeterminate results. Similarly, a positivity rate of 33.8% (135/400) was observed when the ELISA PPE was used. When this latter test was performed 65% (260/400) of samples were non-reactive and 1.3% (5/400) showed indeterminate results. The ELISA EPI and PPE had highly concordant results according to McNemar’s test (*p* = 0.901; Figure 2).

### Nucleic Acid Amplification Tests

NAATs applied to stool samples showed at least a three-fold increase in the schistosomiasis positivity rate compared to KK results. The LAMP assay had the highest positivity rate of the molecular methods of 30.8%, detecting the presence of the parasite DNA in 123 individuals. PCR-ELISA and qPCR also had notably higher positivity rates than KK of 18.5% (74/400) and 16.5% (66/400), respectively (Figure 2). There was evidence of agreement between the results of the PCR-ELISA and qPCR when applying McNemar’s test (*p* = 0.322).

The positivity rate observed using LAMP assay on urine samples was relatively high, although lower than that observed when POC-CCA was used, with positive results for 22% of the study population (89/400). In contrast, the positivity rates observed when either PCR or qPCR were used on the urine samples were much lower, although similar to one another, being 6% (24/400) and 5.2% (21/400), respectively (Figure 2). Accordingly, McNemar’s test only indicated an agreement between the results of the PCR and qPCR tests (*p* = 0.728).

### Chemical analysis of the urine

All urine samples were submitted to chemical analysis using the Combur10 Test® M test strips, enabling detection of leucocytes in the urine from 39 (9.8%), proteins in 36 (9%), hemoglobin in 40 (10%), and nitrite in 19 (4.8%) individuals. For all of these parameters, their absence is the reference value (i.e., that expected in healthy individuals). Alterations in the pH of the urine was detected in 50.3% of the study population, with 36.8% (147/400) of them acidic (pH < 5.5) and 13.5% (54/400) alkaline (pH > 6.5). Multiple separate Fisher’s Exact Tests were used to evaluate the existence of any individual (i.e., bivariate) associations between (i) each of the five urine parameters described above and (ii) the results of each of the four specific diagnostic tests for *S. mansoni* used on the urine samples (i.e., POC-CCA, LAMP, PCR, and qPCR). A significant association was observed only between the presence of protein in the urine and a negative result in POC-CCA (*p* = 0.028).

### Diagnostic test accuracy

The LCA models with two-classes had a smaller BIC statistic compared to models with either one or three classes, therefore the two-classes LCA model was chosen. In this model, class 1 presented higher outcome probabilities for the majority of the tests and was assigned as the ‘case group’ in this study. The outcome probabilities table is available in Supplementary material 3. The estimated prevalence of schistosomiasis using LCA was 13.4%.

In this study, to evaluate the accuracy of the diagnostic tests, only individuals who presented the combination of test outcomes corresponding to probability higher than 80% of being included in Class 1 were classified within the “case group.” As a result, 47 people were considered true positives according to this LCM, with a schistosomiasis prevalence of 12% (95% CI: 9–15%).

LCA was used in this study as the reference standard. Based on this, KK had an estimated sensitivity of 38% and a specificity of 99%. Among all the NAATs methods evaluated in this study, PCR-ELISA, and qPCR, both using stool samples, had the highest estimated values of sensitivity and specificity, being 96 and 92% for PCR-ELISA, and 96 and 94% for qPCR, respectively. Among NAATs performed, the lowest sensitivity rates were observed when using urine samples (6% PCR, 13% qPCR and 19% LAMP). Serological tests had the lowest specificities (67% commercial ELISA kit, 67% ELISA EPI and 69% ELISA PPE), although the commercial ELISA kit also had the highest sensitivity rate (100%). These and the remaining estimated parameters of each diagnostic test evaluated in the current study are listed in Table 3.

**Table 3:** Estimated parameters of the 11 individual diagnostic tests evaluated, and the three 2-step diagnostic approaches proposed for the active search of infected people in moderate and low endemicity areas, in the current study.

Tests	Case (LCA)		Estimated Parameters									Cost/ Sample (USD) **
	**YES**	**NO**	p*	**P**	**sn**	**sp**	**PPV**	**NPV**	**LR+**	**LR-**	**ac**	
POC-CCA + -	27 20	95 258	0.30 LI=0.26 LS=0.35	0.12 LI=0.09 LS=0.15	0.57 LI=0.42 LS=0.72	0.73 LI=0.68 LS=0.78	0.22 LI=0.15 LS=0.31	0.93 LI=0.89 LS=0.96	2.13 LI=1.58 LS=2.88	0.58 LI=0.42 LS=0.82	0.71 LI=0.67 LS=0.76	7.14
LAMP_urine + -	9 38	80 277	0.22 LI=0.18 LS=0.27	0.12 LI=0.09 LS=0.15	0.19 LI=0.09 LS=0.33	0.77 LI=0.73 LS=0.82	0.10 LI=0.05 LS=0.18	0.88 LI=0.84 LS=0.91	0.84 LI=0.46 LS=1.57	1.05 LI=0.90 LS=1.21	0.70 LI=0.66 LS=0.75	2.39
PCR_urine + -	3 44	21 332	0.06 LI=0.04 LS=0.09	0.12 LI=0.09 LS=0.15	0.06 LI=0.01 LS=0.18	0.94 LI=0.91 LS=0.96	0.12 LI=0.03 LS=0.32	0.88 LI=0.85 LS=0.91	1,07 LI=0.33 LS=3.46	1.00 LI=0.92 LS=1.08	0.84 LI=0.80 LS=0.87	2.73
qPCR_urine + -	6 41	15 338	0.05 LI=0.03 LS=0.08	0.12 LI=0.09 LS=0.15	0.13 LI=0.05 LS=0.26	0.96 LI=0.93 LS=0.98	0.29 LI=0.11 LS=0.52	0.89 LI=0.86 LS=0.92	3.00 LI=1.23 LS=7.36	0.91 LI=0.81 LS=1.02	0.86 LI=0.82 LS=0.89	5.14
Kato-Katz + -	18 29	4 349	0.06 LI=0.03 LS=0.08	0.12 LI=0.09 LS=0.15	0.38 LI=0.25 LS=0.54	0.99 LI=0.97 LS=1.00	0.82 LI=0.60 LS=0.95	0.92 LI=0.89 LS=0.95	33.80 LI=11.95 LS=95.60	0.62 LI=0.50 LS=0.78	0.92 LI=0.89 LS=0.94	0.97
LAMP_stool + -	34 13	89 264	0.31 LI=0.26 LS=0.36	0.12 LI=0.09 LS=0.15	0.72 LI=0.57 LS=0.84	0.75 LI=0.70 LS=0.79	0.28 LI=0.20 LS=0.36	0.95 LI=0.92 LS=0.97	2.87 LI=2.23 LS=3.69	0.37 LI=0.23 LS=0.59	0.74 LI=0.70 LS=0.79	2.39
PCR-ELISA_stool + -	45 2	29 324	0.18 LI=0.15 LS=0.23	0.12 LI=0.09 LS=0.15	0.96 LI=0.85 LS=0.99	0.92 LI=0.88 LS=0.94	0.61 LI=0.49 LS=0.72	0.99 LI=0.98 LS=1.00	11.65 LI=8.18 LS=16.60	0.05 LI=0.01 LS=0.18	0.92 LI=0.89 LS=0.95	4.61
qPCR_stool + -	45 2	21 332	0.16 LI=0.13 LS=0.21	0.12 LI=0.09 LS=0.15	0.96 LI=0.85 LS=0.99	0.94 LI=0.91 LS=0.96	0.68 LI=0.56 LS=0.79	0.99 LI=0.98 LS=1.00	16.09 LI=10.58 LS=24.47	0.05 LI=0.01 LS=0.18	0.94 LI=0.91 LS=0.96	5.14
ELISA-EPI + -	15 32	117 236	0.33 LI=0.28 LS=0.38	0.12 LI=0.09 LS=0.15	0.32 LI=0.19 LS=0.47	0.67 LI=0.62 LS=0.72	0.11 LI=0.07 LS=0.18	0.88 LI=0.84 LS=0.92	0.96 LI=0.62 LS=1.50	1.02 LI=0.83 LS=1.26	0.63 LI=0.58 LS=0.68	3.26
ELISA-PPE + -	25 22	110 243	0.34 LI=0.29 LS=0.39	0.12 LI=0.09 LS=0.15	0.53 LI=0.38 LS=0.68	0.69 LI=0.64 LS=0.74	0.19 LI=0.12 LS=0.26	0.92 LI=0.88 LS=0.95	1.71 LI=1.25 LS=2.33	0.68 LI=0.50 LS=0.93	0.67 LI=0.62 LS=0.72	2.70
commercial-ELISA + -	47 0	116 237	0.41 LI=0.36 LS=0.46	0.12 LI=0.09 LS=0.15	1.00 LI=0.92 LS=1.00	0.67 LI=0.62 LS=0.72	0.29 LI=0.22 LS=0.36	1.00 LI=0.98 LS=1.00	3.04 LI=2.62 LS=3.53	-	0.71 LI=0.66 LS=0.75	2.81
Proposition 1 + -	45 2	7 346	0.13 LI=0.10 LS=0.17	0.12 LI=0.09 LS=0.15	0.96 LI=0.85 LS=0.99	0.98 LI=0.96 LS=0.99	0.87 LI=0.74 LS=0.94	0.99 LI=0.98 LS=1.00	48.28 LI=23.13 LS=100.78	0.04 LI=0.01 LS=0.17	0.98 LI=0.96 LS=0.99	4.90
Proposition 2 + -	34 13	32 321	0.16 LI=0.13 LS=0.21	0.12 LI=0.09 LS=0.15	0.72 LI=0.57 LS=0.84	0.91 LI=0.87 LS=0.94	0.52 LI=0.39 LS=0.64	0.96 LI=0.93 LS=0.98	7.98 LI=5.49 LS=11.61	0.30 LI=0.19 LS=0.48	0.89 LI=0.85 LS=0.92	3.78
Proposition 3 + -	45 2	15 338	0.15 LI=0.12 LS=0.19	0.12 LI=0.09 LS=0.15	0.96 LI=0.85 LS=0.99	0.96 LI=0.93 LS=0.98	0.75 LI=0.62 LS=0.85	0.99 LI=0.98 LS=1.00	22.53 LI=13.68 LS=37.11	0.04 LI=0.01 LS=0.17	0.96 LI=0.93 LS=0.98	4.69

LI = limit inferior of the 95% confidence interval; LS = limit superior of the 95% confidence interval; prevalence; p^*^ = positivity rate; p = prevalence; sn = sensitivity; sp. = specificity; PPV = positive predictive value; NPV = negative predictive value, LR+ = positive likelihood ratio; LR– = negative likelihood ratio; ac = accuracy; ^**^ cost/sample column does not include the price of equipment needed, personnel, and DNA extraction kits. The cost of each test was calculated according to the US Dollar exchange rate for 27/07/2022 from the Central Bank of Brazil (https://www.bcb.gov.br/) when 1 USD = 5.3068 BRL.

**Figure 3:**
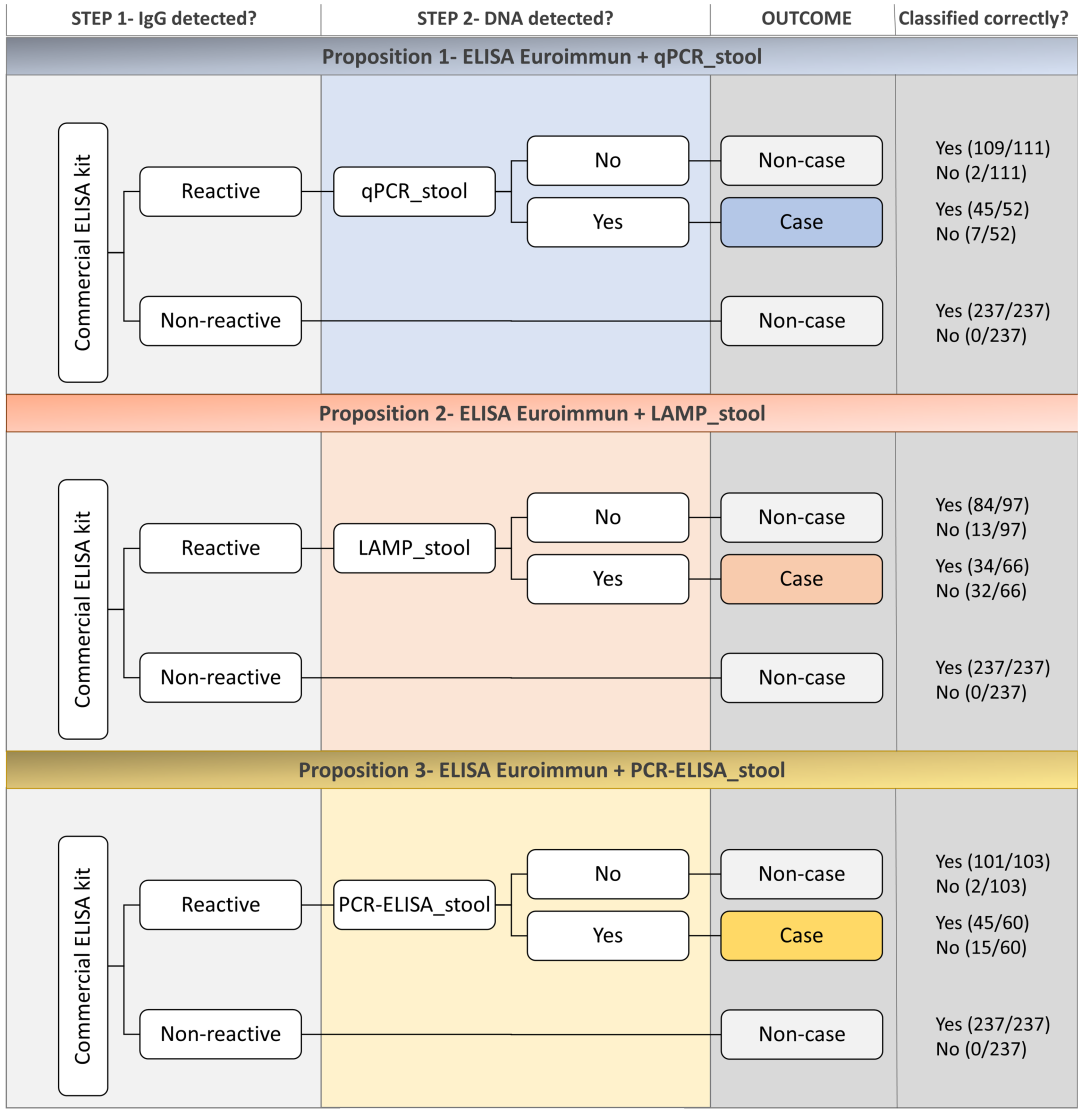
Flowchart illustrating the three different two-step diagnostic approaches proposed for the active search of infected people in endemic areas.

Based on the estimated sensitivity and specificity values of each test (Table 3), three different two-step diagnostic approaches are proposed for the active search of infected people in low and moderate endemicity settings (Figure 3). As the serological commercial ELISA kit had the highest sensitivity among all the tests evaluated, being 100% sensitive, this test is considered an excellent option when screening for true-negative individuals. However, considering the low specificity of this test (67%), it is advised to use a second test with high specificity in order to detect the true-positive among the positive results generated by the commercial ELISA kit. NAATs performed on stool had satisfactory specificity, especially PCR-ELISA and qPCR (92 and 94%, respectively), which are good candidates for the second step of the two-step protocol. Although LAMP had a lower specificity (75%) compared to these two latter tests, it is also considered a candidate for the second step due to its simplicity and feasibility of use in the field.

**Figure 4:**
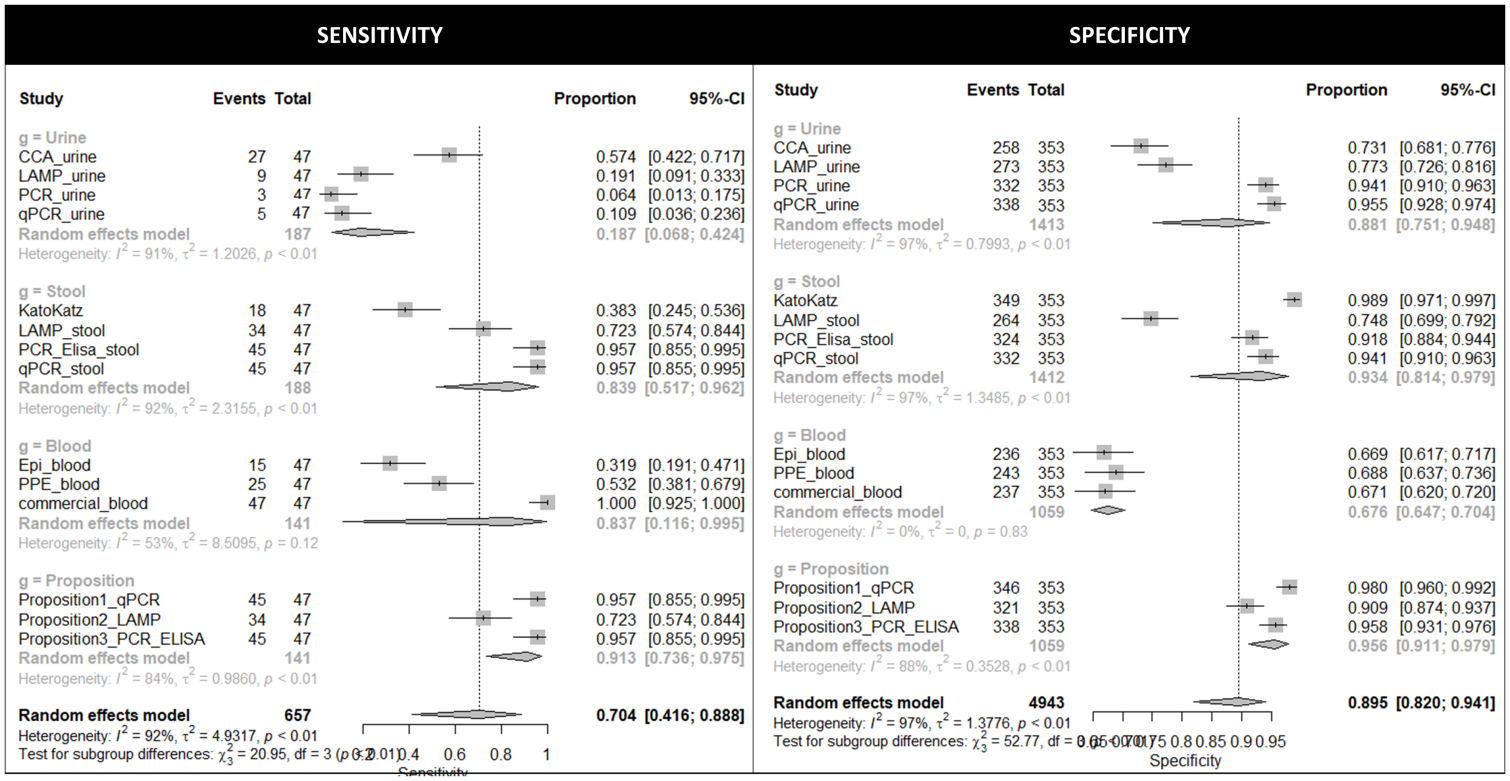
Forest plots illustrating the estimated sensitivity and specificity of the 11 individual diagnostic tests evaluated, and the three two-step diagnostic approaches proposed, in this study.

The estimated parameters for each proposition were calculated and are listed in Table 3. The mean value of sensitivity observed for the 11 tests was 70.4% whilst the specificity was 89.5%, as illustrated in Figure 4. The sensitivity of all three propositions was higher than the general estimated value. Proposition 2 (commercial ELISA kit + LAMP_stool) showed the lowest sensitivity value of 72%, while Propositions 1 and 3 had higher, sensitivity values of 96%. With regard to specificity, all three proposed two-step approaches had similar values ranging from the lowest of 91% (Proposition 2) to the highest of 98% (Proposition 1) and were higher than the general estimated value (89.5%). Propositions 1 (commercial ELISA kit + qPCR_stool) and 3 (commercial ELISA kit + PCR-ELISA_stool) are the most promising candidate methods, with Proposition 1 marginally preferable it has a higher accuracy compared to Proposition 3 (98 and 96%, respectively).

Regarding the costs of each diagnostic test, Kato-Katz had the lower cost (US$ 0.97), followed by LAMP (US$ 2.39), PCR (US$ 2.73), ELISA tests (US$ 2.70–3.26), PCR-ELISA (US$ 4.61), qPCR (US$ 5.14) and POC-CCA (US$ 7.14; Table 3). The two-step diagnosis approach provided similar performance than molecular tests alone with lower costs (Table 3).

## Discussion

There is an urgent need to eliminate schistosomiasis as a public health problem, and precisely diagnosing infected people is vital to achieve this goal. Given the current absence of a standard reference test, and the difficulties of diagnosing the infection in low endemicity areas, we sought to evaluate the performance of 11 diagnostic tests for the detection of *Schistosoma mansoni* infection using the occurrence of infection estimated by latent class analysis (LCA) as the reference standard. LCA is a statistical approach that has been successfully used to assess the accuracy of diagnostic methods for a range of diseases, including schistosomiasis (Ibironke et al., 2012; Colley et al., 2013; Beltrame et al., 2017; Clements et al., 2017; Ferreira et al., 2017; Koukounari et al., 2021). The latent class model (LCM) developed here enabled the estimation of a 12% (95% CI: 9–15%) schistosomiasis prevalence in the borough of Santa Rita, in Malacacheta, Minas Gerais, Brazil. Our findings also enabled the proposition of three different two-step approaches for the accurate diagnosis of *S. mansoni* infection in moderate and low endemicity settings.

We aimed to include a heterogeneous population in our study, with regard to age and gender, in order to enable extrapolation and translation of our findings to other settings. More than 64% of participants reported a past examination for schistosomiasis, with a positive test result for most of them.

Kato-Katz (KK) test is recommended by both the WHO and the Brazilian Ministry of Health (Gomes et al., 2017), for individual diagnosis and epidemiological surveys. In the present study, KK detected *S. mansoni* eggs in stool samples from 22 individuals, with a positivity rate of 5.5%. More than 80% of the KK-positive individuals presented light infections (less than 99 eggs/g). Due to the low sensitivity of KK in low endemicity settings, especially with regard to its ability to detect low intensity infections, it is expected that some infected people have been misdiagnosed as false-negative (McManus et al., 2018; Weerakoon et al., 2018).

In this study, the estimated parameters obtained confirmed that KK has a high specificity (99%) but insufficient sensitivity (38%). This lack of sensitivity is especially critical in regard to control and elimination strategies. According to the WHO, preventive chemotherapy (PC) through mass drug administration (MDA) of praziquantel remains the main strategy for schistosomiasis control and elimination, and to whom PC will be delivered depends on the prevalence of infection in the treated region (Lo et al., 2022). Therefore, timely treatment may be denied to those in need if the true prevalence is underestimated. It is important to reinforce that even low intensity infections are associated with morbidity (King, 2015), as well as parasite cycle maintenance.

Nucleic Acids Amplification Tests (NAATs) are highly sensitive alternatives to microscopic egg detection. Stool samples are usually used as the source of *S. mansoni* genetic material, and a pre-analytical phase is necessary to extract the DNA from eggs and remove inhibitors that can hamper the performance of NAATs. Nucleic acid extraction is an important process, which often requires additional steps to facilitate egg disruption (Pomari et al., 2019). PCR-based methods are more influenced by the presence of inhibitors than isothermal methods (Panzner, 2022). In the present study, qPCR, PCR-ELISA and LAMP were performed using stool samples. PCR-ELISA and qPCR, both targeting the highly repetitive nuclear region termed as *Sm1-7*, had similar positivity rates of 18 and 16%. An agreement was observed between the results of these two methods, probably due to their shared target. The LAMP assay detects the *S. mansoni* mitochondrial minisatellite region (named here *SmMIT*) and had a positivity rate of 31%. Promisingly, PCR-ELISA and qPCR had high sensitivities (96%) and specificity estimates (92 and 94%, respectively). The increased sensitivity of PCR-based methods compared to parasitological diagnosis was expected, in accordance with the findings from previous studies (Espírito-Santo et al., 2014; Siqueira et al., 2015; Senra et al., 2018; do Magalhães et al., 2020; Siqueira et al., 2021; Panzner, 2022). Although highly accurate, qPCR and PCR-ELISA are less field-friendly due to the need for technological equipment, experienced personnel to perform and interpret the results, and the time and costs involved (Li et al., 2021). Isothermal methods have recently become increasingly popular, as promising alternatives for the molecular detection of *S. mansoni* instead of PCR (Fernández-Soto et al., 2014; Gandasegui et al., 2018; García-Bernalt Diego et al., 2019). LAMP is a fast, simple, accurate and field-friendly isothermal method, which can be used at the point-of-care (POC; Notomi et al., 2000; Panzner, 2022). In contrast to previous reports, in this study, LAMP was less sensitive (72%) and specific (75%). Each NAAT assessed in this study has a different limit of detection, with LAMP being able to detect down to 100 fg (Fernández-Soto et al., 2014), PCR-ELISA 1.3 fg (Gomes et al., 2010) and qPCR 0.37 fg (Siqueira et al., 2021). The analytical sensitivity of the assays surely affected the accuracy observed. Given the POC potential of LAMP, future optimization of this assay is advised.

Alternatively, urine samples can also be used for *S. mansoni* detection. A chemical analysis of the urine was conducted to investigate whether the alterations in the standard parameters of the urine influence the outcome of the tests performed using urine samples. Among all the alterations observed, only the presence of protein was associated with a negative outcome of POC-CCA (*p* = 0.028). A previous study assessed variation in urine parameters and their association with the results of POC-CCA, in urine samples where leucocytes were detected it was less expected to observe a positive POC-CCA outcome (Graeff-Teixeira et al., 2021).

The commercially available POC-CCA test detects by lateral flow assay the circulating cathodic antigen released by *S. mansoni* in urine samples. Previous studies have shown that POC-CCA has a higher accuracy when compared to KK in high and moderate endemicity regions (Kittur et al., 2016; Bezerra et al., 2018; Viana et al., 2019). Nevertheless, POC-CCA may give ambiguous results, referred to as ‘trace’, which consists of a weak test line, with no consensus of whether it should be considered as a positive or negative result. Although the manufacturer of this test recommends trace results to be considered positive, there is little evidence of an association between this outcome and the actual presence of infection (Coelho et al., 2016). It has been stated that some illness, use of medicines, and excessive alcohol intake may influence the test result, especially with regard to trace outcomes (Ferreira et al., 2017). Here, 33 individuals were classified as positive by POC-CCA with a clear and strong signal, and 56 trace results. We followed the manufacturer’s instructions and considered the trace results as positives, raising the positive rate from 8.2% (when traces considered negative) to 30% (when traces considered positive). When compared to KK, the POC-CCA test presented a 4-fold increase in positivity. POC-CCA presented a positivity rate of 30%, 3 times higher than the actual prevalence of 12% estimated by LCA. As previously mentioned, the performance of POC-CCA may vary according to the schistosomiasis prevalence and intensity of infection. Besides this, the test is less accurate among people with light infections and in low endemicity areas (Viana et al., 2019; da Ramírez et al., 2020) and may present false-positive results in non-endemic areas (Graeff-Teixeira et al., 2021). The outcome of POC-CCA from Eco Diagnóstica can also be influenced by the batch used (Viana et al., 2019). Furthermore, cross-reactivities have been reported previously due to pregnancy, neoplasia, autoimmune diseases, and other infections, including with soil transmitted helminths (STH; Coelho et al., 2016; Bezerra et al., 2018). However, in the present study, all the individuals positive for STH by KK were POC-CCA-negative.

Besides antigens, urine is also the source of *S. mansoni* cell-free DNA (cfDNA), extracellular DNA fragments which can also be found in serum, and saliva. The origin of cfDNA is still unclear, but it is believed to be released after cellular degradation, apoptosis, or necrosis. The amount of cfDNA present in body fluids is associated with the intensity of parasite infection (Weerakoon and McManus, 2016). The performance of NAATs for cfDNA detection is dependent on accurate selection of the target (Ullah et al., 2021) which can be both the nuclear and/or mitochondrial DNA (Ullah et al., 2022). Several authors described the effective diagnosis of intestinal schistosomiasis by detecting cfDNA in urine and serum samples using different molecular methodologies (Ullah et al., 2022). The presence of *S. japonicum* cfDNA has been detected by PCR in mice as soon as the first week of infection, and by LAMP in rabbits 3 days-post-infection (Gomes et al., 2014; Xu et al., 2015). *Schistosoma haematobium* cfDNA becomes undetectable 2 weeks after treatment with praziquantel (Ibironke et al., 2012). Diagnostic tests based on the presence of eggs in stools are dependent on worm copulation and oviposition, which happens within 40 days-post-infection. Therefore, novel diagnostic methods based on cfDNA may enable the detection of the infection in the prepatent stage of schistosomiasis infection (Ullah et al., 2022). Three NAATs assays were conducted in this study to detect the presence of cfDNA in urine samples, two of them targeting the nuclear *Sm1-7* region and one targeting the *SmMIT* region. In the present study, qPCR and PCR assays targeted the *Sm1-7* region, and had positivity rates of 5 and 6%, respectively, whilst LAMP targeted the *SmMIT* region and had a positivity rate of 22.3%. As observed NAATs using stool samples, methods targeting the same region presented concordant results (i.e., PCR and qPCR; *p* = 0.728). The sensitivity estimates of the urine-based assays were very low, being 19% (LAMP), 13% (qPCR) and 6% (PCR). The unsatisfactory performances observed could be explained by the absence of the target regions in the urine. Nevertheless, previous studies have confirmed that *Sm1-7* (Lodh et al., 2013) and *SmMIT* (Fernández-Soto et al., 2019) markers can be detected in the urine from *S. mansoni-*infected people. Another possible explanation could be some aspect of the pre-analytical stage. However, the clinical urine samples collected here from the study population were processed according to the protocol described by Lodh et al. (2017). Additionally, prior to the application of this protocol on clinical samples, we had successfully validated it in the laboratory using spiked urine samples (data not shown). Given the convenience of urine collection, and the promising results reported by several authors, further investigation should be conducted to optimize the use of cfDNA from urine samples, as a target of NAATs for schistosomiasis diagnosis.

ELISA assays are high throughput tests widely used for the detection of specific antigens and antibodies in blood samples. Immunodiagnostics using antibody detection is more sensitive than KK and particularly useful for monitoring areas of controlled transmission and for the diagnosis of travelers (Cavalcanti et al., 2013). However, they often have low specificity, especially in endemic areas, as they may not be able to differentiate between past and current infection (Weerakoon et al., 2018; Ogongo et al., 2022). It is believed that in endemic areas, all residents will eventually be infected with *S. mansoni* at some point during their lives, thus, it might influence the performance of serological tests in those settings (LoVerde, 2019). The appropriate choice of parasite antigens to be used in ELISA assays is challenging, but vital for the development of accurate tests (Carvalho et al., 2017). The use of recombinant antigens is a promising alternative to increase specificity (Gomes et al., 2014). In the present study, three anti-IgG ELISA tests were conducted, one being a commercially available test (anti-*Schistosoma mansoni* IgG ELISA kit from Euroimmun) and the other two in-house tests: the ELISA EPI using the Smp150390.1 (216–230) peptide, and the ELISA PPE using the recombinant PPE antigens). Carvalho et al. (2017) reported the Smp150390.1 (216–230) peptide as capable of differentiating positive and negative individuals from endemic areas, and negative individuals from non-endemic areas, as well as being less reactive after treatment. Likewise, the recombinant PPE antigen was able to differentiate positive and negative sera (personal communication). The in-house tests employed in the present study provided similar positivity rates of 33% for the ELISA EPI assay and 33.8%, for ELISA PPE, with concordant results. The commercial ELISA kit presented the highest positivity rate of 40.8%. All the serological tests presented low and similar specificity estimates, with commercial ELISA and ELISA EPI kit presenting the lowest (67%), followed by ELISA PPE (69%). Regarding the estimated sensitivity, ELISA EPI was the least sensitive immunoassay (32%) followed by ELISA PPE (53%), while the commercial ELISA kit had a 100% sensitivity, the highest value among all 11 tests assessed. Our results demonstrate that as antigen complexity increases, sensitivity also increases. ELISA EPI test uses as an antigen one epitope from the parasite, ELISA PPE detects the immune response against several epitopes from one parasite protein, while the commercial ELISA kit detects antibody reactivity against epitopes from several proteins of the parasite egg. Therefore, the commercial ELISA kit may be a good candidate test for screening in the absence of the disease, but it will likely provide false-positive results due to either cross-reactivity or detection of past infections.

The recently launched WHO Guidelines on Control and Elimination of Human Schistosomiasis (World Health Organization, 2022) considered the use of a two-step diagnostic approach to detect *S. mansoni* infection, as a strategy for a more accurate diagnosis, especially in low endemicity areas. The combination of multiple tests has been previously evaluated by many authors to increase the accuracy of schistosomiasis detection (Alarcón de Noya et al., 2007; Carneiro et al., 2013; Gomes et al., 2014). We propose in this study three different two-step diagnostic approaches based on the sensitivity and specificity estimates of the 11 individual tests evaluated. The propositions combine the examination of serum samples using the commercial ELISA kit from Euroimmun for the accurate detection of all negative individuals, followed by the molecular detection of *S. mansoni* DNA in stool samples either by (i) qPCR, (ii) LAMP, or (iii) PCR-ELISA, for the accurate detection of true-positive individuals. Overall, the two-step approaches proposed provided similar sensitivity and specificity values observed when using the NAATs alone but with lower costs. Therefore, the cost-effectiveness of the two-step approaches justify their use.

The implementation of novel diagnostic tests is often associated with increased cost. NAATs are usually more expensive than other tests due to the equipment requirement and sample preparation step. The costs associated with the DNA extraction increases substantially the price of each test. For instance, in the present study, the cost for DNA extraction ranged from 8.34 to 16.94 USD for each stool sample. By combining different tests, as suggested in the Proposition 1 (Figure 3), an economy of up to 4,100 USD could be made to examine the whole study population, keeping similar accuracy estimates. Schistosomiasis generates an estimated annual cost of nearly 41 million USD, which could be avoided by properly diagnosing infected people and providing timely treatment with praziquantel (Nascimento et al., 2019). Through these means, besides the economic savings, the health and social burden would also be overcome. Additionally, in the long-term, as the demand for the new products increases the prices tend to decrease (Turner et al., 2017).

Currently, the WHO recommends MDA with praziquantel to every person with 2 or more years of age in areas where the schistosomiasis prevalence is higher than 10% (Lo et al., 2022; World Health Organization, 2022). If KK was the only test result considered, MDA would not be conducted in the study area. The three 2-step protocols proposed in this study have observed prevalence of (i) 13%, (ii) 16%, and (iii) 15%. Therefore, the application of any of these approaches would result in the delivery of PCs to the whole study population regardless of which of the three 2-step protocols were used. Nevertheless, there is evidence that in some low endemicity areas, a relevant hurdle to the MDA strategy is community compliance, and the uptake of praziquantel in the absence of a positive test. In this scenario, the test-and-treat strategy is more targeted and may therefore be more acceptable to the treated population, as well as even more economically justified (Parker et al., 2008; Parker and Allen, 2011; Dabo et al., 2013; Mazigo et al., 2018).

It is believed that the persistence of schistosomiasis transmission is related not only to lack of sanitation and limited access to health care, but also to misdiagnosis of infected individuals with light infections that lead to delayed or absence of timely treatment (da Silva et al., 2022). Thus, to meet the WHO goal of eliminating schistosomiasis as a public health problem by 2030, it is undeniable that diagnostic improvements are necessary and should be prioritized, especially in low endemicity settings.

### Study limitations

The absence of a standard reference test or absolute knowledge of the true positives is a significant limitation, but also the motivation for the research itself. We have overcome this inherent limitation by using LCA to evaluate the diagnostic tests, but the LCA estimated occurrence of infection is influenced by the particular outcome of each test conducted. In addition, this was a cross-sectional study. If a longitudinal study had been conducted, probably a higher prevalence of infection would have been observed given the known daily fluctuation of *S. mansoni* oviposition. Another limitation of our work is the use of three different kits for the DNA extraction from stool samples. The QIAamp DNA Stool Mini Kit has been previously used by members of our team and provided satisfactory results. As production of this kit has been discontinued, we had no choice but to utilize other methods, but this effect was minimized by using two different commercial kits from the same manufacturer. It is reported in the literature that co-infections could influence the outcome of some schistosomiasis diagnosis tests, for instance, HIV impact on the elimination of egg within the feces. However, in this study we did not collected information regarding co-infections other than the ones detected in the Kato-Katz method, therefore we could not address this issue.

## Data availability statement

The original contributions presented in the study are included in the article/Supplementary material, further inquiries can be directed to the corresponding author.

## Ethics statement

The studies involving human participants were reviewed and approved by Ethical Committee of the René Rachou Institute (IRR, Fiocruz Minas), CAAE 76273317.3.0000.5091, and approval numbers 2.400.880, 2.803.752, 3.802.104 and 3.918.849; and (ii) the Ethical Committee of the Oswaldo Cruz Institute (IOC, Fiocruz), CAAE 76273317.3.3001.5248, and approval number 2.426.395. Written informed consent to participate in this study was provided by participants or by the participants’ legal guardian/next of kin.

## Author contributions

RC, TF, CM, LB, MM, EO, RS-P, and CF: conceptualization. SM, RC, TF, CM, LB, GC, FN, GO, LL, MA, EO, RS-P, and CF: methodology and investigation. TS and CF: formal analysis. RC, TF, OC, MM, EO, RS-P, and CF: resources. RC, TF, CM, LB, TS, GC, MM, EO, RS-P, and CF: data curation. SM, TF, CM, LB, TS, EO, RS-P, and CF: writing—original draft. SM, RC, TF, CM, LB, TS, OC, MM, EO, RS-P, and CF: writing – editing. RC, TF, CM, LB, TS, GC, FN, GO, LL, MA, OC, MM, EO, RS-P, and CF: writing—review. SM and TS: visualization. RC and CF: supervision. RC and CF: project administration. RS-P and CF: funding acquisition. All authors contributed to the article and approved the submitted version.

## Funding

This study was financed in part by: René Rachou Institute (IRR-Fiocruz), Oswaldo Cruz Institute (IOC-Fiocruz); Conselho Nacional de Desenvolvimento Científico/Programa de Excelência em Pesquisa—Pesquisa e Ensaios Clínicos (PROEP/PEC; 420685/2017-0); Fundação de Amparo à Pesquisa do Estado de Minas Gerais (FAPEMIG; APQ-01596-16 e APQ-00875-18). SGM is funded by Coordenação de Aperfeiçoamento de Pessoal de Nível Superior-Brasil (CAPES)—Finance Code 001; CAPES Print-Fiocruz Program, and Vice-Presidência de Educação, Informação e Comunicação (VPEIC-Fiocruz); CNPq (Fellowship Grant number: CTF-303131/2018-7; EO-313471/2019-3; MMM—317389/2021-1).

## Conflict of interest

The authors declare that the research was conducted in the absence of any commercial or financial relationships that could be construed as a potential conflict of interest.

## Publisher’s note

All claims expressed in this article are solely those of the authors and do not necessarily represent those of their affiliated organizations, or those of the publisher, the editors and the reviewers. Any product that may be evaluated in this article, or claim that may be made by its manufacturer, is not guaranteed or endorsed by the publisher.
