# Corrigendum: Assessment of the accuracy of 11 different diagnostic tests for the detection of *Schistosomiasis mansoni* in individuals from a Brazilian area of low endemicity using latent class analysis

**DOI:** 10.3389/fmicb.2023.1331715

**Published:** 2023-11-15

**Authors:** Silvia Gonçalves Mesquita, Roberta Lima Caldeira, Tereza Cristina Favre, Cristiano Lara Massara, Lílian Christina Nóbrega Holsbach Beck, Taynãna César Simões, Gardênia Braz Figueiredo de Carvalho, Flória Gabriela dos Santos Neves, Gabriela de Oliveira, Larisse de Souza Barbosa Lacerda, Matheus Alves de Almeida, Omar dos Santos Carvalho, Marina Moraes Mourão, Edward Oliveira, Rosiane A. Silva-Pereira, Cristina Toscano Fonseca

**Affiliations:** ^1^Grupo de Pesquisa em Helmintologia e Malacologia Médica, Instituto René Rachou, Fundação Oswaldo Cruz, Belo Horizonte, Minas Gerais, Brazil; ^2^Laboratório de Educação em Ambiente e Saúde, Instituto Oswaldo Cruz, Fundação Oswaldo Cruz, Rio de Janeiro, Brazil; ^3^Núcleo de Estudos em Saúde Pública e Envelhecimento, Instituto René Rachou, Fundação Oswaldo Cruz, Belo Horizonte, Minas Gerais, Brazil; ^4^Grupo de Pesquisa em Biologia e Imunologia de Doenças Infecciosas e Parasitárias, Instituto René Rachou, Fundação Oswaldo Cruz, Belo Horizonte, Minas Gerais, Brazil; ^5^Grupo de Pesquisa em Genômica Funcional de Parasitos, Instituto René Rachou, Fundação Oswaldo Cruz, Belo Horizonte, Minas Gerais, Brazil

**Keywords:** schistosomiasis, *Schistosoma mansoni*, diagnostic tests, diagnosis, latent class analysis, clinical research, sensitivity, specificity

In the published article, there was an error in Table 1 as published. The sequence of the LAMP internal backward and forward primers (BIP and FIP, respectively) in Table 1 was displayed as BIP 5′ – TTG TGC TCG AGT CGT GGC GCA TAC GAT AGG TGC GAA T-3′ and FIP 5′ ACC GCA GCA TCT CAA TCA AGT CGA AGG CTC CGT CCT AAT – 3′, while the corrected sequences are BIP 5′- AGA AGT GTT TAA CTT GAT GAA GGG GAA ACA AAA CCG AAA CCA CTA−3′and FIP 5′- GCC AAG TAG AGA CTA CAA ACA TCT TTG GGT AAG GTA GAA AAT GTT GT−3′. The correct sequences were added to Table 1 and its caption remains the same as the original table, which appear below.

**Table 1:** Primers and probes used in the molecular tests.

**Type**	**Amplicon**	**Target**	**Assay**	**Sequence**	**References**
Forward primer	121 bp	*S. mansoni* repetitive region *Sm1-7* (GenBank: M61098)	PCR and PCR-ELISA	5-Biosg/GAT CTG AAT CCG ACC AAC CG−3′	Gomes et al., 2009
Reverse primer				5′- ATA TTA ACG CCC ACG CTC TC- 3′	
Probe			PCR-ELISA	5-6[FAM]/TGG TTT CGG AGA TAC AAC GA-3′	
Forward primer	120 bp	Human *β-actin* gene (GenBank: AY582799.1)		5-Biosg/ACC TCA TGA AGA TCC TCA CC-3′	Musso et al., 1996
Reverse primer				5′- CCA TCT CTT GCT CGA AGT CC-3′	
Probe				5-6[FAM]/TCT CCT TAA TGC ACG CAC G-3′	Gomes et al., 2010
Forward primer	90 bp	*S. mansoni* repetitive region *Sm1-7* (GenBank: M61098)	qPCR	5′-CCG ACC AAC CGT TCT ATG A-3′	Siqueira et al., 2021
Reverse primer				5′-CAC GCT CTC GCA AAT AAT CTA AA-3′
Probe				5′-6[FAM]/TCG TTG TAT CTC CGA AACCAC TGG ACG/[3BHQ1]
Forward primer	92 bp	Human *β-actin* gene (GenBank: AY582799.1)		5′-CCA TCT ACG AGG GGT ATG-3′	
Reverse primer				3′-GGT GAG GAT CTT CAT GAG GTA-5′	
Probe				5′- 6[JOE]/CCT GCG TCT GGA CCT GGC TG/[3BHQ1]	
Internal forward primer-FIP	NA	Mitochondrial *S. mansoni* minisatellite DNA region (GenBank: L27240)	LAMP	5′- GCC AAG TAG AGA CTA CAA ACA TCT TTG GGT AAG GTA GAA AAT GTT GT−3′	Fernández-Soto et al., 2014
Internal backward primer-BIP				5′- AGA AGT GTT TAA CTT GAT GAA GGG GAA ACA AAA CCG AAA CCA CTA−3′	
External forward primer-F	203 bp			5′- TTA TCG TCT ATA GTA CGG TAG G-3′	
External backward primer-B3				5′- ATA CTT TAA CCC CCA CCA A-3′	

bp, base pair; FAM, 6-Carboxyfluorescein; BHQ1, Black Hole Quencher 1; JOE, 6-carboxy-4′,5′-dichloro-2′,7′-dimethoxyfluorescein; NA, not applicable.

The authors apologize for this error and state that this does not change the scientific conclusions of the article in any way. The original article has been updated.

## References

[B1] Fernández-Soto P. Gandasegui Arahuetes J. Sánchez Hernández A. López Abán J. Vicente Santiago B. Muro A. (2014). A loop-mediated isothermal amplification (LAMP) assay for early detection of *Schistosoma mansoni* in stool samples: a diagnostic approach in a murine model. PLoS Negl. Trop. Dis. 8, e3126. 10.1371/journal.pntd.0003126 25187956 PMC4154662

[B2] Gomes L. I. dos Marques L. H. S. Enk M. J. de Oliveira M. C. Coelho P. M. Z. Rabello A. (2010). Development and evaluation of a sensitive PCR-ELISA system for detection of Schistosoma infection in feces. PLoS Negl. Trop. Dis. 4, e664, 1–8. 10.1371/journal.pntd.0000664 20421918 PMC2857640

[B3] Gomes L. I. Marques L. H. S. Enk M. J. Coelho P. M. Z. Rabello A. (2009). Further evaluation of an updated PCR assay for the detection of *Schistosoma mansoni* DNA in human stool samples. Mem. Inst. Oswaldo Cruz 104, 1194–1196. 10.1590/S0074-02762009000800021 20140385

[B4] Musso O. Sommer P. Drouet E. Cotte L. Neyra M. Grimaud J. A.. (1996). In situ detection of human cytomegalovirus DNA in gastrointestinal biopsies from AIDS patients by means of various PCR-derived methods. J. Virol. Methods 56, 125–137. 10.1016/0166-0934(95)01892-1 8882643

[B5] Siqueira L. M. V. Senra C. de Oliveira A. A. de Carneiro N. F. Gomes L. I. Rabello A.. (2021). A real-time PCR assay for the diagnosis of intestinal schistosomiasis and cure assessment after the treatment of individuals with low parasite burden. Front. Immunol. 11, 1–13. 10.3389/fimmu.2020.620417 33815351 PMC8010660

