# Supplementary figures and images for: Assessment of the accuracy of 11 different diagnostic tests for the detection of Schistosomiasis mansoni in individuals from a Brazilian area of low endemicity using latent class analysis
Source: Front Microbiol. 2022 Dec 15;13:1048457. doi: 10.3389/fmicb.2022.1048457 (PMC9797737; doi:10.3389/fmicb.2022.1048457)

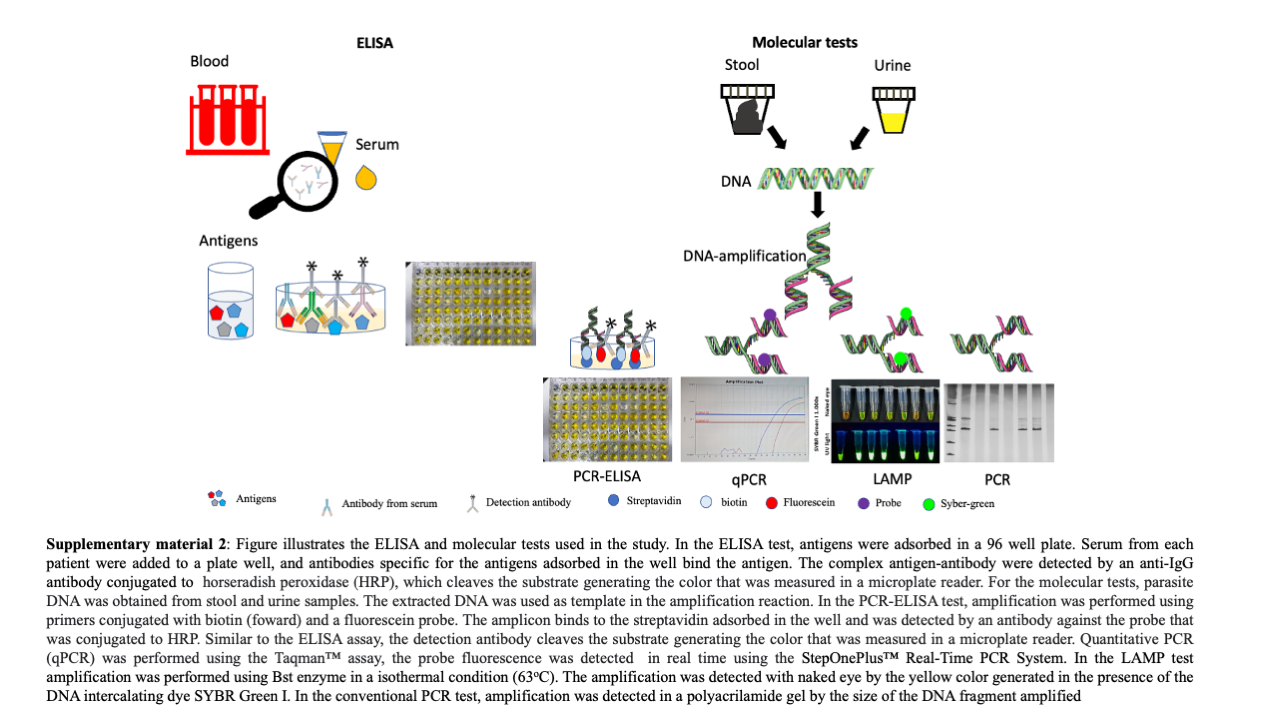

Supplement: Supplementary file 4 [file Image_1.TIFF]
